# Supplementary material for: Content-rich biological network constructed by mining PubMed abstracts
Source: BMC Bioinformatics. 2004 Oct 8;5:147. doi: 10.1186/1471-2105-5-147 (PMC528731; doi:10.1186/1471-2105-5-147)
Supplement: Additional File 2 — The original results of the above study (non-essential files are deleted to keep the file size under the limit set by BMC bioinformatics). [file 1471-2105-5-147-S2.bz2 › chilibotAdditionalFile2/dip40/85ID8702708E312/html/TRAF1_TRAF2.html]

 


 **TRAF1** and **TRAF2** 
  
Found 193 abstracts in PubMed, retrieved 40.  
 

 What does Google say? 
 PDF only 
| .edu only 

---

**Interactive relationship** (e.g. stimulation, inhibition, etc)

**Inhibitory relationship**- **TRAF1**  is unique among TRAFs because it lacks a RING finger domain present in  **TRAF2**  6 that has been shown to be required for  **TRAF2**  and TRAF6 mediated activities.  Ref: 12023442 Sci STKE, 2002
- Thus, expression of this LMP1 domain in  **TRAF1**  positive lymphoma cells promotes significant JNK activation, which is blocked by dominant negative  **TRAF2**  but not TRAF5.  Ref: 12502848 J Virol, 2003
**Neutral relationship**- Regulation of the subcellular localization of tumor necrosis factor receptor associated factor TRAF  [ **TRAF1** ]  2 by  **TRAF1**  reveals mechanisms of  **TRAF2**  signaling.  Ref: 12370254 J Exp Med, 2002
- Here, we show that  **TRAF1**  differentially affects  **TRAF2**  recruitment and activation of NF kappaB by members of the TNF receptor family.  Ref: 12709429 J Biol Chem< MedlineTA>J Biol Chem, 2002
- These findings offer insights into the mechanism of  **TRAF2**  signaling and identify a physiological role for  **TRAF1**  as a regulator of the subcellular localization of  **TRAF2** .  Ref: 12370254 J Exp Med, 2002
- Although c IAP1 bound  **TRAF2**  and  **TRAF1**  in vitro, it ubiquitinated only  **TRAF2** .  Ref: 11907583 Nature, 2002
- Although the  **TRAF2**  TRAF  [ **TRAF1** ]  domain binds ASK1, GCK, and the highly related kinase GCKR, the RING finger domain is needed for their activation.  Ref: 12591926 J Biol Chem, 2003
- The critical role of  **TRAF1**  in the regulation of  **TRAF2**  dependent JNK signaling is particular to the TRAF  [ **TRAF1** ]  binding domain of LMP1, since a homologous region in the cytoplasmic tail of CD40 or the TRADD interacting domain of LMP1 signal on the JNK axis independently of  **TRAF1**  status.  Ref: 12502848 J Virol, 2003
- **TRAF1**  dendritic cells show attenuated responses to secondary stimulation by  **TRAF2**  dependent factors and increased stimulus dependent  **TRAF2**  degradation.  Ref: 12370254 J Exp Med, 2002

**Non-interactive relationship** (e.g. studied together, co-existance, homology, etc.)

- We carried out comparative in situ hybridization analysis of five Traf  [ **TRAF1** ]  genes  **Traf1** ,  **Traf2** , Traf3, Traf4 and Traf6 during murine odontogenesis from the formation of the epithelial thickening to the early bell stage.  Ref: 12711536 Gene Expr Patterns, 2003
- Expression of  **Traf1** ,  **Traf2**  and Traf6 were observed in the outer edges of the bud epithelium whereas Traf3 was strongly expressed at the tip of the bud epithelium.  Ref: 12711536 Gene Expr Patterns, 2003
- Our results suggest that  **TRAF1**  and  **TRAF1**  164 416 exert their regulatory effects on receptor induced NF kappaB activation not only by modulation of  **TRAF2**  receptor interaction but especially  **TRAF1**  164 416 also by directly targeting the IKK complex.  Ref: 12709429 J Biol Chem< MedlineTA>J Biol Chem, 2003
- Western blot analysis further demonstrated that OIP 1 significantly decreased TNF receptor associated factor 2  [ **TRAF2** ]  TRAF  [ **TRAF1** ]  2 expression in RAW 264.7 cells.  Ref: 12619930 J Bone Miner Res, 2003
- To define the role of TRAF  [ **TRAF1** ]  proteins in CD40 dependent isotype switching in B cells, we introduced wild type WT and mutant CD40 transgenes that lacked the binding motifs for TRAF6 CD40deltaTRAF6,  **TRAF2**  and TRAF3 CD40deltaTRAF2 3, or both CD40deltaTRAFs into B cells of CD40 mice.  Ref: 12354380 Immunity, 2002
- However, NFkappaB stimulation by AII differs from that of TNF alpha since a TNF receptor associated factor 2  [ **TRAF2** ]  TRAF  [ **TRAF1** ]  2 dominant negative mutant does not prevent AII mediated NFkappaB activation.  Ref: 12704649 J Cell Physiol, 2003
- We have previously reported that the HCV NS5A protein interacts with TRAF  [ **TRAF1** ]  domain of TNF receptor associated factor 2  [ **TRAF2** ]   **TRAF2** .  Ref: 12796506 J Biol Chem< MedlineTA>J Biol Chem, 2003
- It is proposed that similar adaptive hot spots are also present on the binding surfaces of  **TRAF1** ,  **TRAF2**  and TRAF5.  Ref: 12447905 J Mol Recognit< MedlineTA>J Mol Recognit, 2003
- **TRAF1**  and to a lesser extent  **TRAF2**  were overexpressed in B CLL lymphocytes as compared with normal CD19 B cells.  Ref: 12411322 Blood, 2002
- In this study we demonstrate the ability of the TRAF  [ **TRAF1** ]  binding domain of LMP1 to signal on the JNK AP 1 axis in a cell type dependent manner that critically involves  **TRAF1**  and  **TRAF2** .  Ref: 12502848 J Virol, 2003
- **Traf2**  showed restricted expression in the internal enamel epithelium of the bell stage while expression of  **Traf1** , Traf3, Traf4 and Traf6 were observed in both the internal and the external enamel epithelium.  Ref: 12711536 Gene Expr Patterns, 2003
- The constitutive expression of mRNA for TNF receptors TNFR, including TNFRI and TNFRII, and the adapter molecules, such as the TNF receptor associated death domain protein TRADD, Fas associated death domain protein FADD, receptor interacting protein RIP and TNF receptor associated factor 2  [ **TRAF2** ]  TRAF  [ **TRAF1** ]  2 were analyzed by reverse transcriptase RT PCR in bone marrow samples from control, MDS and AML cases.  Ref: 12681357 Leuk Res, 2003
- Although  **TRAF1**  can displace  **TRAF2**  and CD40 from raft fractions, it promotes the ability of  **TRAF2**  activate signaling over a sustained period of time.  Ref: 12370254 J Exp Med, 2002
